# Antiangiogenic Therapy and Mechanisms of Tumor Resistance in Malignant Glioma

**DOI:** 10.1155/2010/251231

**Published:** 2010-04-11

**Authors:** Ruman Rahman, Stuart Smith, Cheryl Rahman, Richard Grundy

**Affiliations:** ^1^Children's Brain Tumour Research Centre, Medical School, Queens Medical Centre, University of Nottingham, Nottingham NG7 2UH, UK; ^2^Department of Tissue Engineering, Centre for Biomolecular Sciences, University of Nottingham, Nottingham NG7 2RD, UK

## Abstract

Despite advances in surgery, radiation therapy, and chemotherapeutics, patients with malignant glioma have a dismal prognosis. The formations of aberrant tumour vasculature and glioma cell invasion are major obstacles for effective treatment. Angiogenesis is a key event in the progression of malignant gliomas, a process involving endothelial cell proliferation, migration, reorganization of extracellular matrix and tube formation. Such processes are regulated by the homeostatic balance between proangiogenic and antiangiogenic factors, most notably vascular endothelial growth factors (VEGFs) produced by glioma cells. Current strategies targeting VEGF-VEGF receptor signal transduction pathways, though effective in normalizing abnormal tumor vasculature, eventually result in tumor resistance whereby a highly infiltrative and invasive phenotype may be adopted. Here we review recent anti-angiogenic therapy for malignant glioma and highlight implantable devices and nano/microparticles as next-generation methods for chemotherapeutic delivery. Intrinsic and adaptive modes of glioma resistance to anti-angiogenic therapy will be discussed with particular focus on the glioma stem cell paradigm.

## 1. Introduction

Glioblastoma multiforme (GBM) (WHO grade IV astrocytoma) is amongst the most vascular and aggressive of all solid tumors and continues to have an extremely poor prognosis. They are the most common primary brain tumor in adults, with about 4800 new cases each year in the United Kingdom (~17500 per year in the USA), comprising approximately one percent of all tumor diagnoses. The tumor is characterized on histological examination by poorly differentiated neoplastic astrocytes, with cellular polymorphism, nuclear atypia, mitotic activity, necrosis, vascular proliferation and thrombosis [1]. The tumors are highly infiltrative (though distant metastasis is rare) and inevitably recur, even after gross macroscopic surgical resection. However, studies have demonstrated that complete (>90%) resection is associated with survival benefit in both adults [2] and children [3]. Current first choice adjuvant therapy in adults subsequent to maximal surgery is radiation therapy (usually 60 Gy in 30 fractions) with concomitant temozolomide, an alkylating agent. This regime has shown a significant survival benefit [4], extending median survival from 12.1 to 14.6 months. Although a significant improvement, the long-term survival of these patients is still extremely limited with only 8% surviving for four years (compared to zero survivors in the radiotherapy only arm).

In childhood, low-grade astrocytomas are the most common tumor type with high-grade gliomas (HGG) making up 10%–15% of tumors diagnosed [5], a total of around 50–70 cases per year in the U.K. Paediatric central nervous system tumors however account for more expected life years lost than any other tumor group and are now the leading cause of cancer deaths in children, following improvements in survival rates for the leukemias. Treatment broadly follows adult regimes with surgery and radiotherapy being accompanied by alkylating agents. Comparatively, little research has been performed in children regarding therapy and the molecular genetics of high-grade glioma, and although pediatric HGG may resemble adult GBM on histopathological criteria, there are significant differences both clinically and within the molecular biology of the tumors. Long-term survival is more frequent in children, especially those under three years old and it may be that radiotherapy can be avoided in this age group [6]. All modalities of treatment have potentially devastating side effects, often resulting in severe neurological disability. Pathways controlling the generation of new blood vessels (angiogenesis) are frequently implicated in both adult and pediatric tumors. Many genome wide studies have implicated proangiogenic pathways including vascular endothelial growth factor (VEGF) [7], epidermal growth factor (EGF) [8], and platelet-derived growth factor (PDGF) [9]. HGG are in general extremely vascular tumors with evidence of widespread production of new blood vessels. Efforts are currently underway to target these pathways therapeutically with the hope of developing effective novel treatments for HGG that are better tolerated than current cytotoxic chemotherapy. 

It is clear that novel therapies are needed for this tumor to improve the current situation. Here we evaluate current antiangiogenic therapies (summarized in Table 1) and mechanisms of resistance for malignant glioma and consider the development of novel methods of drug delivery to overcome the problem of achieving therapeutic drug concentrations within the CNS caused by the impermeability of the blood brain barrier (BBB).

## 2. Glioma Angiogenesis and Invasion

Glioma vasculature formation occurs through two distinct processes. Glioma angiogenesis is a process involving the genesis of new blood vessels from rerouting and remodeling of preexisting vessels. Neoangiogenesis mainly develops in late embryonic development and during adulthood as a result of tissue demands [10]. Vasculogenesis (blood vessel arrangement) was classically considered an embryonic process but has since been identified in tumors as the *de novo* formation of primitive blood vessels by the differentiation of circulating bone marrow-derived endothelial progenitor cells [11]. Among solid tumors, glioblastoma multiforme displays the most angiogenic features and highest degree of vascular proliferation and endothelial cell hyperplasia [12]. Angiogenesis is thus a key pathologic event in glioblastoma tumors and is necessary for the progression of a localized neoplasm to a highly aggressive tumour. Moreover, malignant gliomas also require angiogenesis to establish a source of nutrients and oxygen and to eliminate cellular waste products [13].

### 2.1. The Molecular and Cellular Angiogenic Switch

Glioma angiogenesis is initiated when the homeostatic balance between pro-angiogenic and anti-angiogenic stimuli is disrupted in favor of the former, resulting in activation of pro-angiogenic signaling pathways. These stimuli are secreted by both cellular sources (glioma cells, endothelial cells and microglia) and environmental triggers (extracellular matrix (ECM), hypoxia) [14, 15]. The transition towards neo-angiogenesis is referred to as the “angiogenic switch” and is increasingly viewed as a rate-limiting secondary event in multistage carcinogenesis [16]. 

The initial stage in the formation of new blood vessels involves the breakdown of native vessels. Angiopoietin-1 (Ang-1) and its receptor Tie-2 are key components of this process and Ang-1 is increased in GBM tumor cells [17, 18]. Glioma cells first accumulate around existing cerebral blood vessels and lift off astrocytic process, leading to the disruption of normal contact between endothelial cells and the basement membrane [18]. Subsequently, these blood vessels become apoptotic and undergo involution. Vascular collapse ensues and results in the death of neighboring tumor cells and the formation of necrotic zones. Hypoxia arises in these regions resulting in expression of hypoxia inducible factor-1 (HIF-1). In GBMs, HIF-1*α* is primarily localized in pseudopalisading cells around areas of necrosis and in tumor cells infiltrating the brain at the tumor margin [19]. HIF-1*α* is a transcriptional master regulator that activates a plethora of genes, the protein products of which function to either increase oxygen availability or to allow metabolic adaptation to oxygen deprivation [20]. In this context, HIF-1*α* transcriptionally activates VEGF which in turn initiates and promotes glioma angiogenesis (see Mediators of glioma angiogenesis below) [21, 22]. 

Following regression of native blood vessels, the basement membrane and surrounding ECM are degraded to allow for endothelial cell invasion. The matrix-metalloproteinases (MMP)-2 and 9 are major factors for this event in brain tumor angiogenesis and MMP-2 and MMP-9 expression is associated with a poor outcome in glioma patients [23, 24]. Upon breakdown of the basement membrane, endothelial cells proliferate and migrate toward tumor cells that express pro-angiogenic factors. Activation of endothelial cells results in upregulation of cell surface adhesion/migration molecules, in particular *α*_*v*_ *β*_3_, *α*_5_ *β*_1_, and CD44 [25].

### 2.2. Pro-Angiogenic Mediators in Glioma

#### 2.2.1. Vascular Endothelial Growth Factor

The VEGF family of growth factors and their receptors are the most important mediators of glioma angiogenesis. VEGF ligands (VEGF-A, VEGF-B, VEGF-C, VEGF-D, and placenta growth factor) bind to and activate the VEGF receptor (VEGFR) tyrosine kinases (VEGFR-1 and VEGFR-2). Specifically, VEGF-A is upregulated in glioblastoma and regulates endothelial cell survival, proliferation, vascular permeability, and migration primarily via VEGFR-2 [26, 27]. VEGF-A is primarily induced by tissue hypoxia via the HIF-1*α* pathway. The hypoxic microenvironment leads to dissociation of von Hippel Lindau protein from HIF-1*α*, preventing its proteasomal-mediated degradation and permitting HIF-1*α* binding to hypoxia response elements in the promoter region of several pro-angiogenic factors, such as VEGF [28]. In addition to HIF-1*α*, a variety of growth factors can also upregulate VEGF expression, including transforming growth factor (TGF)-*β*, EGF, PDGF-B, and basic fibroblast growth factors (FGF) [29–31]. The end result of VEGF signaling in the glioma neovascular niche is the production of immature highly permeable blood vessels with poor maintenance of the blood brain barrier and parenchymal edema [32]. VEGF also functions as a prosurvival factor for endothelia, mediated by suppression of p53, p21, p16, and p27, Bax pro-apoptotic protein and activation of phosphatidylinositol-3 kinase (PI3K)/Akt and Ras/mitogen activated protein kinase (MAPK) pathways [33, 34].

#### 2.2.2. Fibroblast Growth Factor

Similar to VEGF, fibroblast growth factor (FGF) is expressed by glioma cells and their adjacent blood vessels [9]. FGF-receptor 1 is upregulated in endothelial cells while FGF-receptor 4 is expressed primarily in tumor cells [35, 36].

#### 2.2.3. Platelet-Derived Growth Factor

In addition to endothelial cell migration, pericyte (mesenchymal-like cells, associated with the walls of small blood vessels) migration is an important part of the process of tumor vasculogenesis [37]. PDGF secretion by activated endothelial cells recruits pericytes to the site of newly formed vessels and contributes to the development of a new basement membrane [38]. Of the two PDGF tyrosine kinase receptors (PDGF-*α* and PDGF-*β*), PDGF-*α* is expressed in an autocrine manner in glioma cells, whereas PDGF-*β* is expressed in glioma endothelium and pericytes, particularly the latter, suggesting its importance in the migration of pericytes into newly formed blood vessels [39].

#### 2.2.4. Tumor Necrosis Factor

Tumour necrosis factor (TNF)-*α* is a potent inflammatory cytokine found in malignant gliomas and other cells including reactive astrocytes. TNF-*α* induces tumor angiogenesis indirectly via the activation of other angiogenic factors, most notably VEGF [40]. Furthermore, VEGF is upregulated in human gliomas upon TNF-*α* treatment, mediated through the Sp1 transcription factor [41].

#### 2.2.5. Integrins

Integrins are transmembrane receptor molecules that facilitate endothelial cell migration and invasion and specifically, integrin-*α*_*v*_ *β*_3_ correlates to glioma tumor grade and glioma cell proliferation [42, 43].

#### 2.2.6. Matrix Metalloproteinases

MMP-2, and MMP-9 are highly expressed in astrocytomas and correlate with histological grade. Both proteins are detected in tumor and endothelial cells [44]. MMPs are involved in the proteolytic degradation of ECM components and facilitate cell motility during angiogenesis [45]. Upregulation of MMPs is required for the angiogenic effects of TGF-*β* and VEGF and MMP-2 and MMP-9 proteolytically cleave and activate TGF-*β*, thus promoting tumour invasion and angiogenesis [46, 47].

#### 2.2.7. AntiAngiogenic Mediators in Glioma

A number of anti-angiogenic factors have been described and are functionally relevant to tumor angiogenesis. Angiostatin is derived from degradation of plasminogen by proteases such as cathepsin D and MMPs and functions as an endogenous anti-angiogenic factor [48]. Murine models reveal that angiostatin impairs glioma angiogenesis and tumor growth through binding of *α*_*v*_ *β*_3_ on proliferating endothelial cells, resulting in apoptosis [49, 50]. 

The thrombospondins (TSPs) are another family of proteins that function as anti-angiogenic factors. TSP-1 is expressed on platelets, endothelial cells and smooth muscle cells in normal tissue [51]. TSP-1 reduces endothelial cell proliferation and induces apoptosis in vitro [52], TSP peptides derived from TSP-1 decrease glioma angiogenesis and tumor growth in mice [53]. 

Endostatin is a similar anti-angiogenic molecule, formed by proteolytic cleavage of collagen-18 in glioblastoma basement membrane by elastase, cathepsin-L and specific MMPs [54]. Endostatin-mediated angiogenesis blockade includes binding to *α*_5_ *β*_1_ integrin, inhibition of VEFGR-2, and decreased expression of the anti-apoptotic molecule, Bcl-2 [55].

### 2.3. Glioma Invasion

Animal models of glioma invasion fail to accurately mimic the invasiveness of human glioma cells along white matter tracts. Alternative models such as matrigel invasion chambers and xenograft lines serially passaged in vivo have therefore been more informative for study. Glioma cell invasion requires four distinct processes: (i) detachment of invading cells from the primary tumor mass, involving destabilization and disorganization of cadherin-mediated junctions, downregulation of neural cell adhesion molecule and CD44 cleavage which anchors the primary mass to the ECM by the metalloproteinase ADAM; (ii) adhesion to the ECM, mediated by integrins, particularly *α*_*v*_ *β*_3_ which binds fibronectin in the ECM; (iii) degradation of the ECM by proteases such as MMP-2 and MMP-9; and (iv) cell motility and intracellular contractility, mediated by cytoplasmic mediators such as myosin [10]. Invasion along white matter tracts allows gliomas to extend at a microscopic level beyond surgical resection cavities or radiation treatment fields [56]. Glioma cells migrate in a similar fashion to nontransformed neural progenitors, whereby a prominent leading cytoplasmic process is followed by a burst of forward movement by the cell body. This raises the intriguing question of whether stem/progenitor-like cells in glioma have a causative role in glioma invasion, migration and metastases. Indeed glioma stem (or stem-like) cells are highly invasive and are able to invade across the corpus callosum along white matter tracts [57, 58]. Such stem cell models may contribute significantly to future glioma invasion models.

## 3. Anti-Angiogenic Therapies

### 3.1. Antibody Therapies

One of the most well-established anti-angiogenic therapies is bevacizumab (Avastin, Roche). This is an IgG1 monoclonal antibody against free VEGF-A in the circulation, to which it binds, preventing attachment to the VEGF receptor and activation of a pro-angiogenic stimulus. Bevacizumab was originally developed for use in metastatic colorectal and nonsmall cell lung cancers, and has subsequently also been approved by the European Medicines Agency for use in metastatic breast and kidney cancers [59]. In view of the high levels of new vessel formation in GBM, many groups have now used bevacizumab for the treatment of this tumor, often in combination with irinotecan (a topoisomerase inhibitor).

There are currently two phase III trials (NCI and Hoffman La Roche) recruiting newly diagnosed glioblastoma patients for double-blind placebo-controlled studies comparing surgery, radiotherapy and temozolomide with or without bevacizumab, results of which will be of huge interest. 

Phase two trials in patients with recurrent disease have yielded very encouraging results [60] with 6-month progression free survival (PFS) rates of 46% and 6-month overall survival (OS) rate of 77% (*n* = 35 patients) for bevacizumab plus irinotecan in GBM [61]. The same group demonstrated 6-month PFS and OS of 55% and 79%, respectively, for the same agents in recurrent anaplastic astrocytoma (WHO grade III glioma) [62]. Radiological response rates were reported as 57%–63%. Similar response rates have also been observed by other groups in small scale series [63, 64]. As a comparator for this and other trials described, the six-month progression free survival for temozolomide as monotherapy in relapsed GBM has been reported as 21% [65].

Although initial response rates in the desperate situation of recurrent GBM are encouraging, it is clear that this response is temporary, and tumors eventually progress regardless. Attempts to modify bevacizumab regimes with second agents introduced at progression have not proved successful with 6-month PFS of 0%–2% [60, 66]. A particular concern with antibody therapy is its ability to cross the BBB. Clearly bevacizumab may be effective at reducing VEGF levels within the circulating volume, but it has not yet been demonstrated what effect it may have on paracrine VEGF pathways within the tumor and brain parenchyma itself, as it is probably unable to cross the BBB, although this barrier may be deficient in tumors.

Bevacizumab is in general well tolerated with few serious side effects, with approximately 10% of patients having to discontinue treatment in the trials to date. Serious side effects reported include intracerebral haemorrhage, bowel perforation, and thromboembolism. Predicting patients likely to respond to a particular therapy is an area likely to be of increasing significance as more therapies are developed, and more is known of the molecular biology of these tumors. One trial to date has reported that high immunohistochemical VEGF A expression was significantly associated with likelihood of radiographic response, but not overall survival [67]. VEGF single nucleotide polymorphisms may also be indicative of response rates [68].

It has been reported that recurrence after bevacizumab therapy is more likely to be diffuse and distant to the primary tumor location [64, 69]. One possible concern is that the therapy could be inducing increased cell migration within the tumor to escape from areas of hypoxia created by the drug. This diffuse infiltration may not be immediately obvious on conventional gadolinium-enhanced MRI and novel MRI techniques may be required to investigate this phenomenon [70]. It is unclear whether enlarging areas of T2 hyperintensity truly represent tumor invasion or simply increasing edema, with mismatch of clinical and radiological pictures [59].

A similar approach to bevacizumab is VEGF-Trap (Aflibercept—Sanofi/Regeneron). This is a fusion protein soluble decoy receptor with a high affinity for VEGF A [71]. It has been shown in animal glioma models to have significant antitumor activity [72], and phase I/II human clinical trials are underway (NCI).

Early stage trials (NCI) are being undertaken into other monoclonal antibodies directed against PDGFR*α* (IMC-3G3) or VEGFR-2 (ramucirumab), which seems to show promise in early trials in other malignancies [73]. A German randomised controlled trial of nimotuzumab (an EGFR receptor antibody) in newly diagnosed glioblastoma is ongoing. This antibody, along with the similar anti-EGFR antibody cetuximab, has been shown to radiosensitise glioma cells in a mouse model, with nimotuzumab having increased anti-angiogenic and antiproliferative effects [74]. AMG 102 (an antihepatocyte growth factor antibody) has shown promising effects in vitro in combination with an anti-EGFR antibody [75].

### 3.2. Small Molecule Inhibitors

In recent years, there has been much interest in developing inhibitors of various components of the angiogenic pathway. Many of these compounds are of relatively low molecular weight, allowing improved penetration of the BBB and the cytoplasm or nucleus of cells. However, they may still be targeted by drug extrusion systems (e.g., PGP or MDR) and intracellular concentrations may be lowered as a result. In this way, precise molecular targets can be modulated, with the hope of efficacious anti-angiogenic therapy with minimal effect on normal nonangiogenic tissue. The compounds of this type are also usually able to be taken orally, with good tolerability in most trials to date. Agents have been or are in the development process for many molecular targets and they will be summarised here. Many of the small molecule inhibitors act on, for example, multiple molecularly related receptor tyrosine kinases. This may have some advantages in allowing targeting of entire angiogenic pathways, potentially more effective than simply targeting single growth factors or receptors.

### 3.3. Vascular Endothelial Growth Factor Inhibitors

In addition to antibody-based approaches targeting free VEGF, compounds have been developed to target the functioning of the VEGF receptor. An example of this class is cediranib (Recentin/AZD2171—Astra-Zeneca). This is an indole-ether quinazoline that inhibits tyrosine kinase receptors, particularly all subtypes of the VEGF receptor, and has some activity against the PDGF and c-Kit receptors. A phase II trial of cediranib showed a radiological response rate of 56%, with 6-month PFS of 26% in recurrent glioblastoma [59]. The same trial [76] also demonstrated that after just one day of cediranib treatment, magnetic resonance imaging can demonstrate changes in vascular permeability and flow using K^trans^ type techniques. Microvessel volume decreased back towards normal values and these changes were maintained until around the 56 day scan. When the authors combined measures of permeability, microvessel volume, and circulating IV collagen, the “vascular normalisation index” created was an excellent predictor of response and overall survival [77]. The same study also demonstrated reduction in the number of viable circulating endothelial cells and circulating progenitor cells which increased in number when the tumors progressed or relapsed. As well as decreasing new vessel formation, it is hypothesised that the process of normalisation of already formed vessels may enhance the effect of radiotherapy by reducing hypoxia and may enhance the delivery of other chemotherapeutic agents to the tumour. Strategies to extend this window of normalisation before the tumour begins to revert may need to be explored.

Sorafenib (Nexavar—Bayer/Onyx) inhibits a broad range of kinases including serine/threonine and receptor tyrosine kinases. Pathways known to be inhibited by this drug include VEGFR, PDGFR-*β*, c-Kit, and their downstream effectors C-Raf and B-Raf kinases. This in turn leads to decreased MEK 1, 2, ERK and MAPK activity, pathways implicated in cell proliferation as well as angiogenesis [78]. Sorafenib in combination with bortezomib [32] (a proteasome inhibitor) or rottlerin [79] (a protein kinase C inhibitor) has demonstrated efficiency against glioma cell lines, and several phase I/II trials are underway in both newly diagnosed and recurrent GBM. The drug has already been approved for use in renal cell carcinoma, and in common with other anti-angiogenic therapy, hypertension and fatigue are not uncommon side effects. Perhaps of greater concern is the risk of intracerebral haemorrhage which has been reported as higher than usual in patients with renal cell carcinoma cerebral metastases treated with VEGF inhibitors [80].

Sunitinib (Sutent—Pfizer) is an inhibitor of VEGF and PDGF-*β* receptors which has also been used with some success in metastatic renal cell carcinoma. It has demonstrated efficacy in reducing new blood vessel formation, with corresponding survival benefit in both subcutaneous and intracerebral murine glioma models [81] and is currently undergoing phase I/II trials in recurrent GBM in humans. Although a potent inhibitor of angiogenesis, and demonstrating beneficial effects in many animal models, some studies have demonstrated an apparent increase in metastatic behavior of tumors under certain situations, perhaps due to a preconditioning effect on the tumor microenvironment [82, 83]. These findings may echo the increased T2 signal change seen in GBM patients treated with VEGF inhibitors.

AEE788 (Novartis) is an inhibitor of EGFR, and at higher concentrations, VEGFR. This agent had also shown promise in mouse xenograft models when used in combination with everolimus [84], an mTOR pathway inhibitor related to rapamycin (sirolimus). Phase I/II clinical trials are underway currently in recurrent GBM, but further development may be suspended. Other agents being investigated include vatalanib and pazopanib, both VEGF inhibitors with some anti-PDGF activity.

### 3.4. Other Tyrosine Kinase Inhibitors

In addition to the VEGF inhibitors described with anti-PDGF activity, molecules have been developed specifically targeting the PDGF receptor and pathway. The most widely used of these is imatinib (Glivec—Novartis), a small molecule tyrosine kinase inhibitor active against PDGF, Bcr-abl, and c-Kit. Although well tolerated, single agent imatinib had very limited effect against high-grade glioma in adults, particularly in patients on anticonvulsants [85, 86]. Trials are now being undertaken to ascertain whether more effective results can be obtained by the use of imatinib in combination regimes, for example, with vatalanib [87] or temozolomide. Dasatanib, another multikinase inhibitor targeting Src kinases, is currently in early stage clinical trials and has shown promising antiglioma effect in vitro, particularly in combination with temozolomide [88]. NCI phase II studies of another multikinase inhibitor tandutinib are currently ongoing, one in combination with bevacizumab.

Erlotinib (Tarceva—OSI/Roche), is a selective EGFR antagonist that has been used in lung cancer. A phase II study has shown very promising results with median survival of 19.3 months after diagnosis, compared to 14.1 months for historical controls [89], though a phase II trial of its use as monotherapy in recurrent GBM was disappointing [90]. Several further trials are ongoing, including in conjunction with bevacizumab. Gefitinib (Iressa—Astra Zeneca) is another selective EGFR antagonist that binds the ATP binding site of the EGF receptor. Disappointing results in the ISEL lung cancer trial [91] led to it being partially withdrawn in the United States, but hopes remain that a subset of patients with tumours containing appropriate EGF pathway mutations may have some benefit. Studies are ongoing in glioma, especially of its use in combination with other therapy, for example, everolimus [92], though a phase II trial showed limited benefit as monotherapy in recurrent GBM [93].

Other drugs developed to inhibit downstream kinases include enzastaurin (Eli Lilly) a selective protein kinase C *β*-inhibitor which suppresses phosphorylation of many targets including Akt, inhibiting development of xenografted glioma [94]. A phase I trial has showed some promising effects, although dosage was limited by thrombocytopenia and prolonged QT syndrome [95]. The mTOR pathway has also been targeted therapeutically, initially as immunomodulatory therapy for prevention of transplant rejection, but interest in its antineoplastic properties has increased in recent years. This pathway is of interest as it integrates multiple upstream cell signalling mechanisms to regulate protein synthesis, potentially allowing the effects of multiple growth factor receptors to be controlled. Rapamycin (Sirolimus—Wyeth) is a macrolide produced by the bacterium *Streptomyces hygroscopicus *that was originally isolated from soil samples from the island of Rapa Nui (Easter Island). It binds FKBP12 and this combination then inhibits the mTOR pathway. Rapamycin and its analogues temsirolimus and everolimus are currently undergoing phase I/II clinical trials, both as mono- and combination therapy. Temsirolimus has shown promise in published clinical trials, with extended time to progression [96].

### 3.5. Other Agents

Thalidomide (Celgene), a piperidinyl isoindole, has a broad spectrum of anti-angiogenic mechanisms, possibly including suppression of endothelial cell nitric oxide-induced migration, inhibition of TNF*α* and VEGF/IL-6 suppression [97]. In phase II clinical trials, the results have been somewhat mixed, with some groups reporting encouraging outcomes [98, 99], but others showing limited activity [100]. The addition of thalidomide and the cyclo-oxygenase-2 inhibitor celecoxib (shown to decrease angiogenesis in vitro through up regulation of the endogenous inhibitor endostatin) to temozolomide did not improve progression free survival [101]. The use of thalidomide can be limited by adverse effects such as thrombosis or peripheral neuropathy and analogues have been developed such as lenalidomide [102].

Cilengitide (EMD pharmaceuticals) is a cyclic arginine-glycine-aspartic acid peptide that selectively inhibits integrins *α*_*v*_ *β*_3_ and *α*_*v*_ *β*_5_. In a phase II trial, the higher dose regime demonstrated a 6 month PFS of 15% in recurrent GBM [103] and trials are ongoing for its use in combination with temozolomide in newly diagnosed GBM. Other potential targets are the HIFs, which upregulate pro-angiogenic cellular factors in response to low-tissue oxygen partial pressure. The oestrogen derivative 2-methoxyestradiol (2ME2/Panzem—EntreMed) inhibits HIF1*α* mediated VEGF expression and directly downregulates HIF1*α* levels, as well as suppressing microtubule structure formation [97]. Clinical trials of 2ME2 in association with nanocrystal colloidal dispersion are ongoing. Research interest has also focused on attempts to inhibit matrix metalloproteinases (MMPs), important mediators of new vessel growth and tumour invasion. Prinomastat (Agouron) is a hydroxamate-based selective inhibitor of MMPs 2, 9, 13, and 14, which had mixed results in clinical trials in lung cancer [103], with trials in GBM completing at present. Farnesyl transferase inhibitors such as tipifarnib (Zarnestra—Johnson & Johnson) block the farnesylation of the Ras signalling molecule that is needed for it to fulfil its downstream effects in the signalling cascade that occurs after activation of growth factor receptors. Whilst evidence of benefit has been reported in one phase II trial [104], another found no benefit when given as preradiation sensitizer [105].

Histone deacetylase inhibitors (HDACi) are emerging as a promising class of anticancer agent, which act by alleviating transcriptionally silenced pathways in tumors, such as tumor suppressor pathways. HDACi have also been shown to possess antiinvasive and anti-angiogenic potential with SAHA/vorinostat currently undergoing phase II trials for recurrent GBM [106–108]. Although it is unclear whether these compounds have anti-angiogenic effects in glioma, we have revealed anti-proliferative and pro-apoptotic effects using the HDACi Trichoststin A in pediatric glioblastoma cells (R. Rahman, manuscript in preparation).

### 3.6. Pediatric Tumours

Very few trials of anti-angiogenic therapy have been performed in pediatric high-grade glioma. As previously stated, this is unfortunate as it is increasingly clear that it may not be possible to extrapolate results from adult trials to pediatric practice as genome wide studies indicate that there may be key differences in the molecular biology of HGG in different age groups.

Chemotherapy administered to children has often been performed in a metronomic fashion with the intention of targeting the tumor endothelium, and studies utilising thalidomide have been undertaken, with a subset of patients having prolonged PFS [109]. A phase I study of cilengitide demonstrated good response in patients completing the treatment and a phase II study is planned. Erlotinib has been evaluated in a phase I trial in children, adolescents, and young adults with newly diagnosed HGG [110], with evaluation proceeding. A phase I trial of imatinib in children with newly diagnosed brainstem and recurrent malignant gliomas showed some concerns with intratumoral haemorrhage and dose limiting toxicity in patients with brain stem glioma but seemed well tolerated in recurrent glioma [111]. Tipifarinib has been used in a phase I trial in brainstem glioma [112] and a phase II trial in several high-grade brain tumours with good tolerability but little effect [90]. A phase I trial of semaxanib (SU5416) was terminated early due to the sponsor ceasing development of the drug, although prolonged disease stabilisation was seen in 25% of patients given the higher dosage [113].

## 4. Mechanisms of Glioma Anti-Angiogenic Resistance

**Figure 1:**
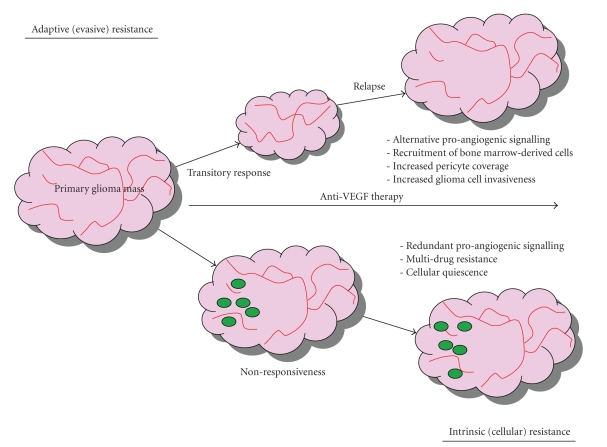
Mechanisms of glioma resistance to anti-angiogenic therapeutic modalities. *(Top) Adaptive (evasive) resistance. *After an initial transitory response phase, the tumor switches to mechanisms that induce neovascularization and renewed tumor growth and progression, thereby evading therapeutic blockade. These consist of pro-angiogenic factor substitution (typically dependence on FGF and angiopoietin signalling in cases of VEGF blockade), recruitment of endothelial cells and pericytes from the bon-marrow, protection of existing tumor blood vessels via increased pericyte coverage and increased tumor cell invasiveness whereby tumor cells invade adjacent normal tissue to achieve vascular sufficiency. *(Bottom) Intrinsic (cellular) resistance. *From the outset, some gliomas are nonresponsive to angiogenesis blockade. This may be accounted for by the preexistence of multiple redundant pro-angiogenic signals, which would allow for continued angiogenesis during anti-angiogenic insults. In addition, glioma stem cells (GSCs) (*green circles*) have been identified as key mediators of glioma angiogenesis and may share intrinsic cell survival and lifespan prolonging characteristics with normal tissue stem cells. Specifically, GSCs may reside in a noncycling quiescent state, thus blocking entry of drugs through the tumor cell membrane and may express relatively high levels of ABC-drug transporters, enabling a multi-drug resistance phenotype via efflux of drugs from the tumor cell. *Red lines, tumor blood vessels. *

Benefits of anti-angiogenic therapy both in preclinical settings and to patients are at best transitory, typically in the form of tumor stasis or shrinkage and in few cases, increased survival. Inevitably this period of clinical benefit (measured in weeks/months) is followed by restoration of tumor growth and progression [114]. Indeed, inhibiting VEGF does not appear to block tumour progression. Such resistance to anti-angiogenic therapy is counter-intuitive to the proposition that angiogenesis is essential for the progression of malignant glioma. Knowledge of the mechanistic basis governing anti-angiogenic resistance is required to fine-tune and better specify future treatment protocols using these drugs. The current status quo proposes two general modes of resistance to angiogenesis inhibitors, particularly those targeting VEGF and related pathways: adaptive (evasive) resistance, and intrinsic (preexisting) resistance (Figure 1) [115]. Multiple mechanisms are likely to underlie both modes of resistance. 

### 4.1. Adaptive (Evasive) Resistance

An evolving hypothesis is that angiogenic tumors acquire the means to functionally evade the angiogenesis blockade induced by angiogenesis inhibitors [116–119]. Evasive resistance is indirect in so much as alternative means to sustain tumor growth are activated but the specific therapeutic target of the anti-angiogenic agent remains inhibited (Figure 1, top) [119]. Activation and/or upregulation of alternative pro-angiogenic signaling pathways may be one distinct adaptive mechanism, whereby substitution of a pro-angiogenic factor reestablishes neovascularization [120]. Evidence for FGF-dependent revascularization has come from a clinical investigation of glioblastoma patients being treated with the VEGFR inhibitor cediranib. After a measureable response phase, a relapse/progression phase was associated with higher blood levels of FGF2 compared to the same patients during the response phase [76]. Evaluation of the prevalence of this mechanism in human gliomas would be greatly facilitated by studying tissue obtained from patients undergoing re-resection subsequent to recurrence after anti-angiogenic therapy.

Recruitment of vascular progenitor cells and pro-angiogenic monocytes from the bone marrow is another distinct mechanism of resistance. Anti-angiogenic therapy-induced blood vessel regression may lead to hypoxia, creating conditions permissive for the recruitment of a heterogeneous population of bone marrow-derived monocytic cells that promote angiogenesis [115]. Specifically, these cells consist of endothelial and pericyte progenitors which differentiate into endothelial cells forming the inner lining of blood vessels, or pericytes that envelop blood vessels, respectively [121]. In GBM, HIF-1*α* recruits various pro-angiogenic bone marrow-derived CD45+ myeloid cells, and tumors lacking HIF-1*α* exhibit few such cells and are severely impaired in their angiogenic and tumor growth phenotypes [122]. These studies provide a mechanistic rationale for how hypoxic tension can create an environment that promotes neovascularization. 

Although inhibition of VEGF signaling pathways causes vessel regression, a few thin vessels remain, densely and tightly covered with pericytes. Protective coating by pericytes presumably helps the tumor endothelium to survive and grow during the course of any anti-angiogenic therapy regime [121]. The contribution of this mode of resistance in GBM is undefined at present.

The switch to a condition of increased invasiveness without angiogenesis is another method of evasive adaptation. This phenotype was first described in orthotopic GBM mouse models, where neovascularization was blocked by genetically deleting VEGF and HIF-1*α*. GBM cells coopted normal blood vessels (perivascular invasion) to achieve the required vasculature in a dispersed fashion [123].

### 4.2. Intrinsic (Cellular) Resistance

A considerable minority of GBM patients tested in clinical trials for bevacizumab, sorafenib, and sunitinib failed to show even transitory clinical benefit [76]. As no period of tumor stasis was evident, these tumors are refractory to angiogenic therapy. It is plausible that the preexistence of FGF2 and other pro-angiogenic factors in late-stage GBM tumors could enable continued angiogenesis using redundant pathways during anti-angiogenic treatment. 

Of particular interest is the glioma cell-type that is nonresponsive to anti-angiogenic therapy (and other chemotherapeutics). The stem cell paradigm for malignant gliomas presents tumour-initiating events as occurring within the genome of a cellular entity with intrinsic or acquired stem/progenitor cell-like properties. Glioma stem cells (GSC) have been reported to promote angiogenesis and vasculogenesis via increased expression of VEGF and stromal-derived factor 1 [124, 125]. Tumors enriched for GSCs showed increased vessel density, increased endothelial cell proliferation and tubule formation, increased endothelial progenitor mobilization, and recruitment of bone marrow-derived cells [125]. Although it may be counter-intuitive to expect GSCs, which by definition represent a very small minority of glioma tumor cells, to make a meaningful contribution to glioma angiogenesis, it is important to note that during early tumor-initiation or the seeding of a metastatic lesion, the GSC fraction would constitute a much greater proportion of the tumor mass. Therefore, it is conceivable that GSCs provide the necessary signals to trip the angiogenic switch early during the tumor growth of primary and/or metastatic tumors. Moreover, the hypoxic microenvironment has recently been shown to promote the expansion of GSC populations and promote a more stem-like phenotype in nonstem cell glioma populations [126, 127]. 

Regardless of whether the glioma cell of origin is a tissue-specific stem/progenitor cell or the specificity of cell surface antigens in delineating tumor-initiating glioma cells, the evidence that a subpopulation of glioma cells shares certain cardinal properties of stem cells and early progenitors (namely capacity for self-renewal and multi-lineage differentiation) provides a conceptual and technical framework in which to understand cellular resistance to therapies such as anti-angiogenic agents. It is logical to query whether surviving glioma cells intrinsically resistant to angiogenic therapy share characteristics with normal tissue stem cells which permit a long lifespan, such as cellular quiescence, expression of ATP-binding cassette (ABC) drug transporters, and increased DNA repair capacity. 

Malignant gliomas that are resistant to chemotherapeutics often display a multidrug resistance phenotype due to reduced cellular drug accumulation through ABC membrane efflux pumps [128]. In addition, tissue stem cells generally reside in the G0 stage of the cell cycle and are only induced to activate self-renewal and differentiation programs when the respective tissue needs to be repopulated. It is possible that the expression of ABC transporters and the quiescent state of malignant GSCs may be key determinants of intrinsic nonresponsiveness to anti-angiogenic therapies, by the extrusion of agents from the cell, and by providing a barrier for entry of agents into the cell, respectively (Figure 1, bottom). In addition, studies have shown that GSCs promote radioresistance by preferential activation of the DNA damage response [124]. 

A number of caveats for these hypotheses emerge however. The expression profiles of drug resistance-related ABC transporters did not differ between primary and secondary glioblastomas and no correlation to recurrent tumors was evident [128]. Moreover, glioma cell populations sorted for the expression of the ABCG2-transporter, revealed that both ABCG2+ and ABCG2− populations exhibited similar tumorigenicity [129]. Regarding the noncycling nature of GSCs, direct evidence for the prevalence of quiescence in glioma is lacking. Through studies of stem cell and proliferation marker expression coupled with telomerase enzymatic activity, we find evidence for lower proliferation levels in childhood ependymoma cell populations enriched for stem cells (R. Rahman, unpublished findings). However we cannot discriminate between a general reduced rate of proliferation in the population as a whole or to the existence of a quiescent subset within the total population. It remains to be elucidated whether the aforementioned modes of intrinsic GSCs are necessary and sufficient to achieve intrinsic resistance in malignant glioma. The relationship between the hypoxic glioma microenvironment and GSCs may also impact upon future therapy as it has recently been shown that HIF proteins are preferentially activated in GSCs compared to nontumor cells [130]. Better understanding in this respect will aid development of novel agents specifically targeting GSCs.

## 5. Future Therapeutic Directions

The question of how to prolong the sometimes excellent shortterm results of anti-angiogenic therapy is the subject of much investigation. Current trials are focusing on combination approaches using multiple therapies to target different pathways concurrently with the hope of preventing the use of different pathways to evade the inhibition of a single molecule. Novel agents are also underdevelopment which will target the previously discussed pathways more effectively, and also with the aim of controlling mechanisms not yet fully elucidated.

One promising new field that may yield effective means of controlling the angiogenic process is that of microRNAs. These are short (20–23 base pair) conserved sequences that are transcribed but not translated. First identified in *C. elegans* in the early 1990s, it is only in the last 10 years that their true importance has been recognized [56]. They act by binding complementary sequences on messenger RNA and interfering with (usually downregulating) the translation of the mRNA into protein. There are now several hundred recognised miRNA known to regulate human gene translation, with approximately 50% of cancer related genes having known miRNA regulators. High-grade gliomas have characteristic profiles of miRNA expression [131–133], and the differences between HGG, low-grade gliomas, and normal tissue may give important clues as to how these short RNA sequences control tumor growth and development, including angiogenesis [134]. In other brain tumors such as medulloblastoma [135], particular miRNAs have been shown to be closely linked to the molecular machinery driving the tumor. Various miRNA [136–138] have been shown to be over or underexpressed in adult HGG with changes in the particular miRNA identified profoundly influencing glioma growth. miRNA has been shown to be crucial to normal brain development [139, 140] and to the processes of blood vessel creation and angiogenesis in normal development [141] as well as in pathological processes such as tumors. It has also been shown in glioma cell lines that the upregulation of VEGF and other factors in response to hypoxia may be governed by miRNA expression levels [142]. 

Therapeutically, it has been demonstrated that the effect of a particular miRNA can be strongly inhibited by engineered oligonucleotide sequences, so-called “antagomirs”, with consequent decreased levels of related protein synthesis [143]. It has been demonstrated in a murine liver cancer model that dramatic reduction in tumor burden can be achieved by reactivating expression of miR-26a which is down-regulated in liver cancer [144]. In human brain tumors it has been shown that miR-296 is elevated in tumor-related endothelial cells of new vessels and that this miRNA may govern growth factor receptor expression [145]. It seems likely that much work will focus on elucidating which are the key miRNAs in GBM, how to antagonise/upregulate them and on the delivery vectors that would be necessary to use them in clinical trials [146]. A recent in vitro report showed good activity against CD133+ glioma stem cells by a combination of imatinib and the miRNA 451 [147]. Complete dispersion of neurospheres was observed at low concentrations of the combined reagents.

### 5.1. Polymeric Controlled Release for Intracranial Drug Delivery

Over the past two decades, a variety of approaches to enhance intracranial chemotherapeutic drug delivery have been investigated. Local therapies (via injection) are diffusion limited and may not reach areas distant to the site of injection. One approach is polymeric-controlled release for direct delivery of agents to intracranial tumors. The rationale of such an approach is to improve upon the efficacy and reduce the debilitating side effects of current systemic chemotherapeutics. An attractive feature of biodegradable polymers is that they completely erode during drug delivery and are cleared from the body. The majority of biodegradable polymers undergo erosion simply by water permeating into the polymer matrix [148]. 

There are two main methods of utilising polymeric controlled release for intracranial drug delivery: implantable devices and nano- or microparticles for injection. Implantable biodegradable polymeric devices provide a practical means of localizing the chemotherapeutic agents specifically at the tumor site. An additional advantage of direct intracranial drug delivery is that the need for a chemotherapeutic agent to cross the BBB is eliminated. 

The most common copolymer system used intracranially is polybis (p-carboxyphenoxy) propane-sebacic acid (p (CPP-SA)). This delivery system has been characterized for a variety of drugs and is in clinical use [149]. Many controlled release systems are based on an implantable wafer. The p(CPP-SA) wafer loaded with 1,3-*bis* (2-chloroethyl)-1-nitrosourea (BCNU), also known as carmustine, is available clinically as Gliadel [150]. Gliadel is implanted intracranially after surgical debulking of the tumor. It is commonly used for local delivery of BCNU to high-grade gliomas after resection and is associated with increased survival [151]; however drug diffusion from the site of implantation is limited.

Implantable poly(lactic-co-glycolic acid) (PLGA) matrices loaded with chemotherapeutics are currently under development [149]. In an analogous method to Gliadel, the use of biodegradable PLGA wafers containing BCNU has been investigated and research in this area is ongoing [152]. Biodegradable polymer matrices based on polymers of lactide and glycolide are a popular platform for local drug delivery. PLGA particles have been used as a controlled delivery system for proteins, drugs, cytokines, hormones, enzymes, vaccines, and chemotherapeutic agents [153–156]. The composition of the PLGA allows the control of the degradation rate and therefore the control of drug release kinetics [157].

In addition to controlled release via wafers, polymers can also be harnessed to aid delivery of chemotherapeutic drugs to the brain by injection of polymeric nano- or microparticles. To date, minimally invasive systemic delivery of drugs to the brain remains a challenge that has given rise to the development of new drug-targeting technologies. Many forms of systemic chemotherapy are excluded from the central nervous system by the BBB and the high systemic concentrations necessary to cross the BBB often lead to several side effects [158, 159]. The use of nano- or microparticles for drug delivery to the brain appears to be a promising option to overcome these problems.

The advantage of polymer particles lies in the route of administration. Nano- or microparticles may be injected stereotactically to any site in the brain due to their size and spherical shape [160]. This is less invasive than the implantation of polymeric wafers [149]. Recently, BCNU-loaded PLGA microspheres were developed in vitro for intracranial administration by cerebral stereotaxy, which could potentially be administered repeatedly [161]. The use of PLGA particles to deliver drugs other than BCNU is also being investigated. Benny and colleagues demonstrated the delivery of two endogenous anti-angiogenic inhibitors, hemopexin (PEX) and a fragment of platelet factor 4 (PF-4) (PF-4/CTF), in vitro and in vivo for human glioma therapy using PLGA microspheres, with glioma growth inhibition observed.

Over the last decade, coating polymeric particles with a surfactant has also been investigated to enhance intravenous delivery of chemotherapeutic drugs to the brain. As evidenced by a number of studies, poly(butyl cyanoacrylate) (PBCA) nanoparticles coated with polysorbate 80 (Tween 80) facilitate brain delivery of a number of drugs that are unable to cross the BBB in free form [162]. Polysorbate 80 also proved to be effective for brain delivery of different types of nanoparticles such as poly (alkyl cyanoacrylate) and solid lipid nanoparticles [163, 164]. Polysorbate 80-coated PBCA nanoparticles have been found to selectively adsorb certain plasma proteins from the blood. These proteins promote receptor-mediated endocytosis of the polymer particles by the endothelial cells forming the BBB, thus facilitating delivery of the nanoparticle-encapsulated drug to the brain [165].

The effectiveness of the polysorbate 80-coated PBCA nanoparticles for brain delivery was most clearly demonstrated by the high antitumor effect of nanoparticle-bound doxorubicin against intracranial glioblastoma in rats [166]. A different type of surfactant, poloxamer 188 (Pluronic F68), was also found to be effective for brain delivery by PBCA particles [167, 168]. Recently, Gelperina and colleagues showed that both polysorbate 80 and poloxamer 188 were also able to facilitate brain delivery for PLGA nanoparticles [169]. 

Harnessing polymeric controlled release in the form of implants and injectable particles is proving to be a promising area of investigation for intracranial drug delivery. Ongoing and future research into this innovative drug delivery method will strive to address the need for controllable intracranial drug delivery for glioma therapy.

## 6. Summary and Perspectives

Although the transitory efficacy of anti-angiogenic inhibitors such as those targeting VEGF signalling pathways are disappointing, with a temporary period of response followed by relapse, these results must be considered in the context of standard of care therapy for malignant glioma, in particular GBM. These strategies also typically exhibit an initial response followed by inevitable resistance and tumor progression. Also, the majority of patient trials have involved recurrent glioma tumors at a late-stage of disease progression. Studies of anti-angiogenic therapies in patients with newly diagnosed malignant glioma will help determine whether these agents have greater efficacy in an earlier setting. 

Approaches to overcome anti-angiogenic resistance may include more potent anti-angiogenic agents, synergistic strategies with drugs that inhibit other relevant targets, or multi-targeted single agents that simultaneously inhibit several crucial targets. The optimum protocol for the use of anti-angiogenic therapy in combination with cytotoxic chemotherapy and radiation therapy is as yet unclear for gliomas and needs to be refined. The concept of a “window” for increased therapeutic effectiveness, opened by anti-angiogenic therapy through normalization of vasculature, is also being explored and may impact on regimes adopted in future trials. Combination therapy with drugs that target glioma invasion is a promising approach due to concerns that anti-angiogenic therapy may lead to infiltrative tumor growth. Furthermore, future modes of chemotherapeutic delivery such as stereotactic injection of nanoparticles and tumor site-specific implants concomitant with surgical resection may circumvent the inefficiency and reduce the toxicity of systemic drug delivery and lead to a more targeted and minimally invasive therapeutic approach to glioma. 

Increasing evidence for the persistence of stem cell-like cells in glioma may shift therapeutic approaches to target these cell populations. With the elucidation of the role of GSCs in glioma angiogenesis, it is tempting to speculate that much of the observed intrinsic resistance and non-responsiveness to anti-angiogenic therapies is due to inherent properties of GSCs. We await a generation of cytotoxic agents that specifically target glioma cells with stem cell/progenitor properties. Combination strategies with such compounds and anti-angiogenic drugs may be an exciting future avenue, where the latter mode of therapy chemosensitizes the tumor prior to a direct cytotoxic hit on GSCs. 

A number of pertinent questions remain to be better understood, answers of which will influence future modes of therapy: are GSCs enriched in regions of hypoxia so prevalent in GBM tumors? Is the cell type resistant to anti-angiogenic therapy, the same cell type resistant to chemotherapy and/or radiation therapy? What distinguishes anti-angiogenic glioma cells with adaptive resistance and those with intrinsic resistance? Can the same glioma cell type within a given tumor exhibit both modes of anti-angiogenic resistance and what are the implications for therapy if so? What are the roles of bone marrow-derived cells and stromal cells in the process of anti-angiogenic resistance? Are GSCs involved in invasion, migration, and metastases and are such cells the same GSCs that initiated the primary tumor?

Research into glioma tumor biology will be required to identify novel targets amenable to anti-angiogenic therapy. In conjunction, the development of novel methods for chemotherapeutic delivery will continue to advance our understanding of targeting tumor angiogenesis with wider application for other solid tumors. Disruption of glioma neovascularisation not only remains an attractive method of intervention but provides a model to better overcome glioma resistance.

## Figures and Tables

**Table 1:** Anti-angiogenic agents trialled in high-grade glioma and their respective targets.

*Antibody Therapies*	*Major Molecular Targets*
Bevacizumab	Free VEGF-A
IMC-3G3	PDGFR alpha
Ramucirumab	VEGFR-2
Nimotuzumab	EGFR
AMG-102	Hepatocyte Growth Factor
VEGF-trap	Decoy receptor for VEGF

*Tyrosine Kinase Inhibitors*	
Cediranib	VEGFR/PDGFR/c-Kit
Sorafenib	VEGFR/PDGFR/c-Kit/Raf
Sunitinib	VEGFR/PDGFR
AE788	EGFR/VEGFR
Erlotinib	EGFR
Imatinib	PDGFR/Bcr-abl/c-Kit
Dasatanib	Src kinases

*Signal Pathway Inhibitors*	
Enzastaurin	Protein Kinase C
Rapamycin/Temsirolimus	mTOR
Tipifarnib	Ras

*Other Agents*	
Thalidomide/Lenalidomide	NO/TNF alpha/IL-6
Celecoxib	Endostatin
Cilengitide	Integrins *α*_*v*_ *β*_3_ and *α*_*v*_ *β*_5_
2-methoxyestradiol	HIF1*α*
Prinomastat	MMPs 2,9,13 and 14
SAHA(Vorinostat)	Histone deacetylase
